# Increased Frequencies of Myeloid-Derived Suppressor Cells Precede Immunodiscordance in HIV-Infected Subjects

**DOI:** 10.3389/fimmu.2020.581307

**Published:** 2020-11-06

**Authors:** Isaac Rosado-Sánchez, Rebeca De Pablo-Bernal, Anna Rull, Juan Gónzalez, Santiago Moreno, David Vinuesa, Vicente Estrada, María Ángeles Muñoz-Fernández, Francesc Vidal, Manuel Leal, Yolanda María Pacheco

**Affiliations:** ^1^ Institute of Biomedicine of Seville (IBiS), Virgen del Rocío University Hospital (HUVR)/Consejo Superior de Investigaciones Científicas (CSIC)/University of Seville, Seville, Spain; ^2^ Universitat Rovira i Virgili, Instituto de Investigación Sanitaria Pere Virgili (IISPV), Hospital Universitari de Tarragona Joan XXIII, Tarragona, Spain; ^3^ Unidad de Enfermedades Infecciosas, Servicio de Medicina Interna, Hospital Universitario La Paz, Madrid, Spain; ^4^ Servicio de Enfermedades Infecciosas, Hospital Ramón y Cajal, Madrid, Spain; ^5^ Unidad de Enfermedades Infecciosas, Hospital Universitario, Universitario San Cecilio, Granada, Spain; ^6^ Hospital Universitario Clínico San Carlos, Madrid, Spain; ^7^ Molecular Immunology Laboratory, Hospital General Universitario Gregorio Marañón, Madrid, Spain; ^8^ Health Research Institute Gregorio Marañón (IiSGM), Spanish HIV HGM BioBank, Madrid, Spain; ^9^ Networking Research Center on Bioengineering, Biomaterials and Nanomedicine (CIBER-BBN), Madrid, Spain; ^10^ Internal Medicine Service, Hospital Viamed Santa Ángela de la Cruz, Seville, Spain

**Keywords:** HIV, immunodiscordance, MDSC, monocytes, Th17, Treg, CCR2, PDL-1

## Abstract

**Background:**

We have previously observed increased levels of inflammatory biomarkers and Th17 as well as Treg cells, but not other T-cell specific alterations, preceding immunodiscordance of successfully-treated HIV-infected subjects. Our hypothesis is that this could be related with potential alterations in myeloid-derived suppressor cells (MDSCs) and/or monocyte subsets.

**Methods:**

We determined the frequencies of MDSCs and monocyte subsets and the expression of several functional markers (CCR2, *β*7-integrin, IDO, PDL1, CD11b) in HIV-infected subjects before treatment. We additionally analyzed follow-up samples after 24 months of suppressive cART in a subgroup of subjects. Bivariate regressions were performed, and correlations with soluble proinflammatory and bacterial translocation biomarkers, as well as with Th17/Treg ratio and anti-CMV titers were explored.

**Results:**

Increased frequencies of MDSCs, but normal distribution of monocyte subsets, preceded immunodiscordance. The expression of several functional markers, such as CCR2, CD16, CD11b and PDL1, on MDSCs and monocyte subsets was altered in this scenario. MDSC and monocyte-related functional markers were associated with soluble biomarkers and T-cell parameters. Several of these cellular alterations were not restored after 24 months of suppressive cART.

**Conclusion:**

An early immunosuppressive environment, characterized by the expansion of MDSCs and Tregs, precedes immunodiscordance and is related with a highly inflammatory status.

## Introduction

Although the combined antiretroviral therapy (cART) usually suppresses HIV viremia to lead to a rise in CD4 T-cell counts (1), a proportion of subjects persistently maintain low CD4 T-cell counts (immunodiscordant subjects to cART) (2). These subjects show high rates of non-AIDS complications and death (3); however, current therapeutic approaches aimed at increasing their CD4 T-cell counts or at improving their clinical outcome have failed thus far (4), encouraging research on the subjacent mechanisms. Among them, the potential involvement of the innate immune cells in immunodiscordance has been barely explored.

Myeloid-derived suppressor cells (MDSCs) are intermediates of normal myeloid differentiation (5). They constitute a group of suppressive immature cells with heterogeneous phenotypes: monocytic-MDSCs (m-MDSCs) and granulocytic-MDSCs (g-MDSCs) (5). Although MDSCs are present at low levels in healthy individuals, they are expanded in chronic progressive HIV-1 infection (6–8). Recently, it has been described that such expansion occurs very early at infection (9, 10), suggesting that it could affect to the CD4 T-cell recovery along treatment (10). Importantly, the HIV-driven expansion of m-MDSCs has been shown to promote the differentiation of Tregs (11).

Monocytes are also relevant innate cells able to produce proinflammatory mediators and to impact on differentiation of several T-cell subsets, such as Tregs and Th17 cells (12). Monocytes are well-known contributors to the pathogenesis of HIV infection (13). Three monocyte subsets with different phenotypes and functions have been described: classical (CD14^high^CD16^-^), intermediate (CD14^high^CD16^+^) and patrolling (CD14^low^CD16^high^) (14). A few reports about monocytes and immunodiscordance exist, showing an increase in intermediate populations with increased expression of activation markers in subjects who experienced a poor CD4 recovery in response to cART (15–17).

During the last years, we have described severe immunological alterations preceding immunodiscordance in a deeply characterized cohort of subjects with an incomplete CD4 recovery after treatment. This cohort has been comparatively studied with a control group of adequate CD4 recovery being matched by baseline CD4 T-cell counts (18–20). These early/baseline alterations included increased proliferation of CD4 T-cells, increased frequencies of Th17 cells and regulatory T-cells (Tregs) as well as IL17-producing Tregs, and increased levels of proinflammatory mediators, such as IL-6 and C-reactive protein (hsCRP). In contrast, we observed that the expression of markers for T-cell activation (HLA-DR), senescence (CD28-CD57+) or exhaustion (PD-1) did not precede the immunodiscordance. Our aim herein was to explore also innate components as MDSC and monocytes in this cohort of immunodiscordant subjects, in baseline and in post-treatment samples.

## Methods

### Study Subjects

Samples of HIV-infected subjects were selected from the Spanish AIDS Research Network Cohort (CoRIS) (21) and provided by the HIV BioBank of the Spanish AIDS Research Network (RIS) (22). CoRIS provided clinical data, although hematological parameters, as total leukocytes or CD8 T-cell counts, had not been routinely collected in the database at the time of study, preventing from calculate absolute MDSCs numbers and CD4/CD8 T-cell ratio. The flow chart of selection criteria has been described elsewhere (18). Briefly, we selected pre-cART samples from two different groups of antiretroviral-naïve HIV-infected subjects who had started cART with <200 CD4/mm^3^: one group achieving less than 250 CD4/mm^3^ after 24 months of suppressive cART (late-treated subjects with low CD4 recovery; LR-subjects), and a control group achieving more than 250 CD4/mm^3^ after 24 months of suppressive cART (late-treated subjects with high CD4 recovery; HR-subjects). Finally, 21 samples from each group, matched by sex, age, viral load and baseline CD4 counts, were originally selected (18). As a result of a hierarchical strategy, samples from 13 LR- and 15 HR-subjects were available for the analyses presented herein. Additional available post-cART follow-up samples (24 ± 6 months on cART) from both HIV groups (six LR- and five HR-subjects) were also analyzed.

### Flow Cytometry and Immunophenotyping

Peripheral blood mononuclear cells (PBMCs) were thawed and stained with surface antibodies, followed by fixation and permeabilization according to the manufacturer’s instructions (Foxp3/Transcription Factor Staining Buffer, Ebioscience), and subsequently stained with intracellular antibodies. The antibodies and fluorochromes used are described in **Supplementary Methods**. Viable cells were identified using LIVE/DEAD fixable Aqua Blue Dead Cell Stain (Life Technologies, USA). After exclusion of T-cells (CD3^+^), B-cells (CD19^+^ and/or CD20^+^) and NK cells (CD56^+^), total-MDSCs and monocytic-derived MDSCs (m-MDSCs) were defined as HLA-DR^−^CD33^+^CD11b^+^ and HLA-DR^−^CD33^+^CD11b^+^CD14^+^, respectively, while granulocytic-derived MDSCs (g-MDSCs) were defined as CD11b^+^CD15^+^CD14^−^. Monocyte subsets were defined as classical monocytes (HLA-DR^+^CD14^high^CD16^−^), intermediate monocytes (HLA-DR^+^CD14^high^CD16^+^) and patrolling monocytes (HLA-DR^+^CD14^low^CD16^high^). We also identified monocytes that suffered shedding of CD14 and CD16 (HLA-DR^+^CD14^low^CD16^low/−^). A schematic diagram of the gating strategy used is shown in **Supplementary Figure 1**. Expression of homing molecules, such as *β*7-integrin and chemokine-receptor 2 (CCR2), activation and suppressive molecules, such as CD11b and programmed death-ligand 1 (PDL1), respectively, and metabolic enzymes, such as indoleamine 2,3-dioxygenase (IDO), were determined in these cellular subsets. Moreover, the intensity of CD16 expression was also determined in the monocyte subsets. Regulatory T cells (Tregs) were defined as live CD3^+^CD4^+^CD25^high^Foxp3^+^. Th17 cells were defined as CD3^+^CD4^+^ T-cells able to produce IL17A after stimulation with 2.5 µg/ml of phorbol-12-myristate 13-acetate (PMA) and 1 µg/ml of ionomycin during 12 h. Isotype controls were used when necessary. The expression of functional molecules was only determined on cellular subsets with more than a hundred events. Flow cytometry was performed on an LSR Fortessa (BD, USA), and the data were analyzed using FlowJo version 9.2 (Tree Star). The data are always expressed as frequencies (%), with the exception of CD16 and CD11b expression in monocyte subsets, which are expressed as the Mean Fluorescence Intensity (MFI).

### Soluble Markers

IFN-*γ*-inducible protein 10 (IP-10) and IL-6 was quantitated using 4-plex panels (Bio-Plex, Bio-Rad Laboratories, USA). High-sensitivity C-reactive protein (hsCRP) and D-dimer levels (HemosIL D-Dimer HS 500, USA) were determined using automated clinical chemistry analyzers. Soluble hyaluronic acid (HA) (R&D, USA), soluble CD14 (sCD14) (Diaclone, France), and anti-CMV immunoglobulin G (IgG) titers (Abnova, Taiwan) were quantitated according to the manufacturer’s instructions. Lipopolysaccharide (LPS) was analyzed as previously described (19).

### Statistical Analyses

Continuous variables are expressed as the median and interquartile range (IQR), whereas categorical variables are expressed as numbers and percentages (%). The Mann–Whitney *U* and Wilcoxon rank tests were used to analyze unpaired and paired comparisons, respectively. Correlations were assessed using the Spearman rank test. Bivariate regressions were performed for adjustment by CD4 T-cell counts at cART initiation. The results were expressed as odds ratios (ORs) and 95% confidence intervals (CIs). A *p*-value <0.05 was considered statistically significant. Prism, version 5.0 (GraphPad Software, Inc.) and Statistical Package for the Social Sciences software (SPSS 21.0, USA) were used for the generation of graphs and for statistical analysis, respectively.

## Results

### Clinical Characteristics of the Studied Subjects

Samples from 28 HIV-infected male subjects (13 LR- and 15 HR-subjects) before cART initiation and follow-up samples from 11 of these subjects (six LR- and five HR-subjects) were available for this study. The clinical characterization of the two groups at cART onset showed no differences in age, viral load, sexual transmission or previous C event, whereas a tendency toward lower CD4 T-cell counts was observed in LR-subjects **(** **Table 1** **)**. After 24 months on cART, CD4 T-cell counts were 207 [147–239] and 429 [362–502] in the LR- and HR-subjects, respectively.

**Table 1:** Clinical Characteristics of the studied HIV-infected subjects before cART onset.

	LR-subjects (13)	HR-subjects (15)	*p*
**Age (years)**	43 [32–57]	40 [33–49]	0.5
**CD4 counts at baseline sample**	70 [53–116]	136 [72–165]	0.104
**Log Viral load before cART**	4.9 [4.1–5.3]	4.9 [4.4–5.7]	0.8
**Sexual transmission; n/n (%)**	10/13 (77)	12/15 (80)	0.8
**Previous C event; n/n (%)**	4/13 (31)	3/15 (20)	0.5

Continuous variables are expressed as the median and interquartile range [IQR], whereas categorical variables are expressed as the number and percentage (%). The Mann–Whitney U test was used for comparisons.

### Before cART, LR-Subjects Showed Higher Frequencies of Myeloid-Derived Suppressive Cells (MDSCs) With Higher Expression of CCR2 and PDL1

**Figure 1:**
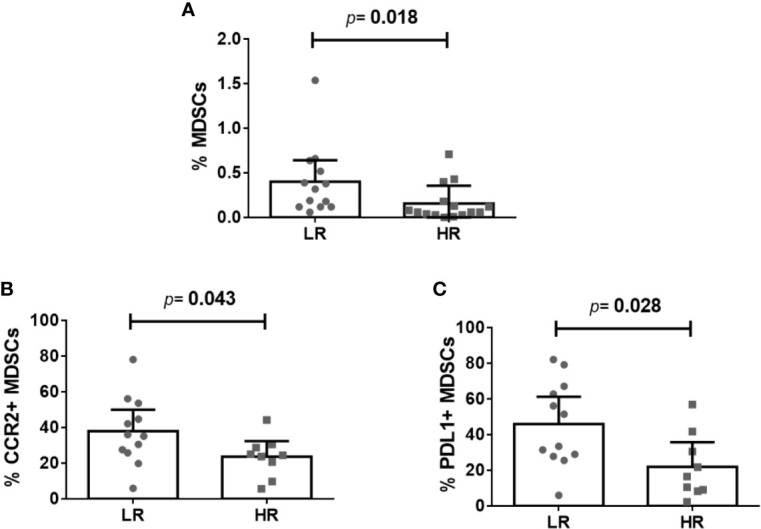
Myeloid-derived suppressor cells (MDSCs) and their expression of functional markers preceding immunodiscordance. **(A)** Frequency of total-MDSCs, **(B)** frequency of CCR2 + MDSCs, and **(C)** frequency of PDL1 + MDSCs in LR- and HR-subjects. The Mann–Whitney *U* test was used for comparisons. If excluding the highest value of MDSCs in the LR group **(A)**, p = 0.032.

We analyzed the frequencies of total-MDSCs, m-MDSCs, and g-MDSCs as well as the expression of different functional markers on MDSCs (**Table 2**). However, the extremely low number of events obtained of g-MDSCs prevented us from accurately determining this MDSC population (data not shown). Remarkably, we observed increased frequencies of total-MDSCs (*p* = 0.018) (**Figure 1A**) and m-MDSCs (*p* = 0.020) in LR-subjects before cART initiation, as well as increased frequencies of CCR2^+^MDSCs (*p* = 0.043) (**Figure 1B**) and PDL1^+^MDSCs (*p* = 0.028) (**Figure 1C**). No difference was observed in the frequencies of IDO- or *β*7-positive MDSCs. Similar trends were obtained after adjustment for CD4 T-cell counts (**Table 2**).

**Table 2:** Myeloid-derived suppressive cells (MDSCs) and cellular markers before cART initiation.

	LR-subjects (13)	HR-subjects (15)	*P*	Univariate AnalysisOR (CI); *p*	Bivariate AnalysisOR (CI); *p*
**% total-MDSCs**	0.3 [0.1–0.6]	0.1 [0.0–0.2]	**0.018**	42.32 (0.76–23158.11); *0.068*	21.06 (0.37–1251.30); *0.138*
**% m-MDSCs**	0.2 [0.1–0.5]	0.1 [0.0–0.3]	**0.020**	60.35 (0.53–6831.94); *0.089*	26.30 (0.20–3422.43); 0.188
% CCR2^+^ MDSCs*	35.6 [26.3–51.5]	24.5 [15.2–29.8]	**0.043**	1.07 (0.99–1.16); *0.084*	1.06 (0.98–1.15); *0.141*
% *β*7^+^ MDSCs*	53.8 [19.5–73.1]	54.0 [10.0–88.7]	0.9	1.01 (0.97–1.03); 0.9	0.99 (0.97–1.03); 0.9
% IDO^+^ MDSCs*	2.0 [0.0–33.7]	3.4 [0.0–69.2]	0.6	0.98 (0.95–1.01); 0.3	0.98 (0.95–1.01); 0.3
% PDL1^+^ MDSCs*	42.5 [28.2–66.1]	16.6 [8.7–36.3]	**0.028**	1.06 (1.00–1.12); **0.042**	1.06 (1.00–1.12); **0.045**

Variables are expressed as median and interquartile range [IQR]. p, Mann–Whitney U test was used for comparisons. A bivariate regression was performed in order to adjust by CD4 T-cell counts at cART initiation. Results from regression analyses were expressed as odds ratios (ORs) and 95% confidence intervals (CIs). *These comparisons were performed in 21 subjects (LR = 12, HR = 9). Statistically significant values are highlighted in bold, p < 0.005.

Longitudinal analyses showed that cART increased the frequencies of CCR2^+^ MDSCs in both groups, although reaching statistical significance only in LR-subjects (*p* = 0.046), as well as the total frequencies of MDSCs only in HR-subjects (*p* = 0.043) (**Table 3**). Only marginal variations in the frequencies of CCR2- and *β*7-positive MDSCs were observed in HR-subjects. A transversal comparison of post cART samples did not show any difference in these parameters between post-cART samples (**Table 3**).

**Table 3:** Follow-up analysis of MDSCs and cellular markers (24-months of suppressive cART).

	LR-Subjects (6)			HR-Subjects (5)			
	Before cART	Post cART	*pW*	Before cART	Post cART	*pW*	*pM-W*
**% total-MDSCs**	0.3 [0.1–0.6]	0.1 [0.1–0-4]	0.3	0.1 [0.0–0.1]	0.2 [0.0–0.2]	**0.043**	0.9
**% m-MDSCs**	0.3 [0.1–0.4]	0.1 [0.1–0.3]	0.3	0.1 [0.0–0.2]	0.1 [0.0–0.2]	0.5	0.9
% CCR2^+^ MDSCs*	30.6 [16.4–40.4]	48.1 [27.5–58.8]	**0.046**	25.1 [23.9–25.1]	50.3 [40.7–50.3]	*0.109*	0.9
% *β*7^+^ MDSCs*	60.4 [26.0–72.2]	45.6 [19.5–69.2]	0.6	14.8 [10.3–14.8]	9.4 [8.5–9.4]	*0.109*	0.5
% IDO^+^ MDSCs*	2.0 [0.0–28.9]	3.0 [0.6–7.8]	0.5	3.4 [0.0–3.4]	4.1 [0.0–4.1]	0.180	0.8
% PDL1^+^ MDSCs*	42.5 [28.7–62.7]	32.5 [19.6–65.6]	0.5	16.6 [9.2–16.6]	22.8 [8.9–22.8]	0.9	0.3

Variables are expressed as median and interquartile range [IQR]. pW, Wilcoxon rank test was used for longitudinal comparisons. pM-W, Mann–Whitney U test was used for transversal comparisons of post cART data. *The expression of functional molecules was determined in nine subjects (LR = 6 and HR = 3). Statistically significant values are highlighted in bold, p < 0.005.

### Before cART, LR-Subjects Showed No Differences in the Frequencies of Monocyte Subsets, but Trends of Differential Expression of Functional Markers

**Figure 2:**
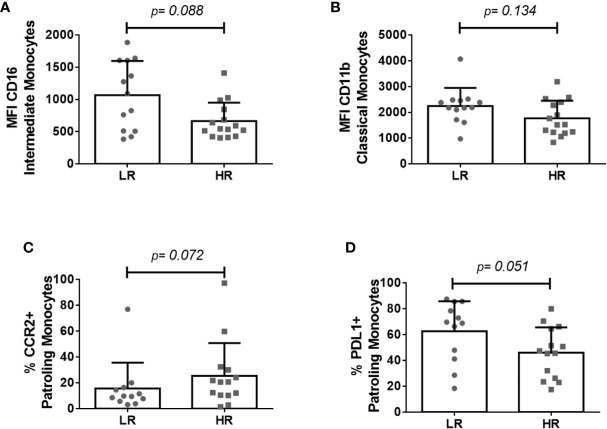
Monocyte subsets and their expression of functional markers preceding immunodiscordance. **(A)** CD16 levels on intermediate monocytes, **(B)** CD11b levels on classical monocytes, **(C)** frequency of CCR2^+^ patrolling monocytes, and **(D)** frequency of PDL1^+^ patrolling monocytes in LR- and HR-subjects. The Mann–Whitney *U* test was used for comparisons. MFI, Mean Fluorescence Intensity.

We also analyzed the frequencies of monocyte subsets and the expression of different functional molecules. No difference in any monocyte subset was observed between groups before cART onset (**Table 4**). However, LR-subjects showed a trend toward a lower frequency of shedding monocytes whereas toward higher level of expression of CD16 in different monocyte subsets (**Figure 2A**). Moreover, tendencies toward higher levels of CD11b in classical monocytes (**Figure 2B**), lower frequencies of CCR2^+^ intermediate and patrolling monocytes (**Figure 2C**), and higher frequencies of PDL1^+^ classical and patrolling monocytes (**Figure 2D**) were observed in LR-subjects. Similar results were obtained after adjusting for CD4 T-cell counts (**Table 4**).

**Table 4:** Monocyte subsets and expression of different functional molecules before cART initiation.

	LR-subjects (13)	HR-subjects (15)	*p*	Univariate AnalysisOR (CI); *p*	Bivariate AnalysisOR (CI); *p*
**% Classical monocytes**	79.6 [65.6–88.1]	69.7 [62.0–84.9]	0.3	1.03 (0.98–1.03); 0.2	1.03 (0.97–1.09); 0.4
**MFI CD11b**	2,189 [1,910–2,469]	1,521 [1,228–2,399]	*0.134*	1.01 (1.00–1.00); *0.094*	1.01 (1.00–1.00); *0.118*
**% CCR2**	82.8 [74.4–90.8]	84.2 [78.2–89.9]	0.9	0.98 (0.93–1.05); 0.6	0.97 (0.90–1.0); 0.3
**% *β*7**	9.1 [4.1–26.2]	16.7 [2.6–26.9]	0.9	0.99 (0.96–1.03); 0.6	0.98 (0.94–1.02); 0.3
**% IDO**	1.4 [0.6–3.4]	1.0 [0.7–4.6]	0.9	1.05 (0.83–1.22); 0.9	0.98 (0.98–1.20); 0.8
**% PDL1**	23.9 [12.3–42.3]	13.7 [7.0–31.6]	*0.062*	1.04 (0.99–0.11); *0.114*	1.03 (0.99–1.08); 0.163
**% Intermediate monocytes**	10.8 [6.6–13.7]	15.4 [9.1–26.1]	0.3	0.97 (0.91–1.04); 0.4	0.97 (0.90–1.04); 0.4
**MFI CD16**	1,090 [511–1,607]	544 [429–845]	0.088	1.02 (1.00–1.00); **0.030**	1.02 (1.00–1.00); *0.054*
**MFI CD11b**	2,281 [1,944–3,052]	2,395 [1,324–2,956]	0.4	1.00 (0.99–1.00); 0.4	1.00 (0.99–1.00); 0.4
**% CCR2**	51.9 [44.3–61.4]	64.8 [45.9–80.0]	0.189	0.97 (0.93–1.02); 0.189	0.94 (0.91–1.01); *0.097*
**% *β*7**	9.1 [2.5–16.4]	6.9 [2.5–24.3]	0.7	0.98 (0.93–1.03); 0.4	0.96 (0.91–1.01); *0.123*
**% IDO**	3.5 [1.0–7.8]	1.9 [0.7–6.8]	0.5	1.03 (0.92–1.17); 0.6	1.02 (0.89–1.15); 0.8
**% PDL1**	47.7 [31.0–69.5]	37.4 [23.6–58.7]	0.2	1.02 (0.98–1.05); 0.3	1.01 (0.98–1.05); 0.5
**% Patrolling monocytes**	2.8 [1.9–8.9]	4.1 [2.4–7.5]	0.6	1.01 (0.86–1.16); 0.9	1.09 (0.91–1.31); 0.4
MFI CD16*	2,372 [1,307–3,253]	1,248 [872–2,513]	*0.123*	1.00 (1.00–1.00); 0.3	1.00 (1.00–1.01); 0.3
MFI CD11b*	827 [612–1,029]	734 [512–1,007]	0.6	1.00 (0.99–1.00); 0.8	0.99 (0.99–1.00); 0.4
% CCR2*	9.6 [6.2–15.9]	20.6 [10.4–30.6]	*0.072*	0.98 (0.94–1.02); 0.3	0.97 (0.93–1.02); 0.189
% *β*7*	5.4 [4.1–10.4]	8.8 [4.9–27.3]	0.2	0.96 (0.90–1.02); 0.2	0.94 (0.88–1.01); *0.079*
% IDO*	0.4 [0.1–1.4]	0.5 [0.3–1.4]	0.7	1.08 (0.74–1.59); 0.7	1.04 (0.70–1.53); 0.9
% PDL1*	69.3 [42.9–83.9]	46.5 [25.6–64.9]	*0.051*	1.04 (0.99–1.08); *0.069*	1.03 (0.99–1.08); 0.166
**% Shedding monocytes**	4.3 [2.5–5.2]	5.9 [3.7–9.9]	*0.072*	0.69 (0.48–1.01); *0.058*	0.71 (0.48–1.05); *0.088*

Variables are expressed as median and interquartile range [IQR]. p, Mann–Whitney U test was used for comparisons. A bivariate regression was performed in order to adjust by CD4 T-cell counts at cART initiation. Results were expressed as odds ratios (ORs) and 95% confidence intervals (CIs). *Comparisons of the expression of molecules were performed in 26 subjects (LR = 12, HR = 14). Statistically significant values are highlighted in bold, p < 0.005.

Longitudinal analysis showed no reduction of CD11b levels in LR-subjects, but a tendency toward a decrease in HR-subjects (*p* = 0.08) (**Table 5**). Moreover, HR-, but not LR-subjects increased the levels of CD16 in intermediate monocytes (*p* = 0.043), whereas LR-subjects increased the frequency of shedding monocytes (*p* = 0.028). Moreover, LR-subjects significantly reduced the frequencies of IDO^+^ monocyte subsets (*p* = 0.028 for classical and intermediate subsets and *p* = 0.046 for patrolling monocytes), whereas HR-subjects only showed tendencies toward a decrease or even no change in this marker on cART. Finally, both groups significantly increased the frequencies of CCR2^+^ classical monocytes but decreased the frequencies of PDL1^+^ patrolling monocytes (*p* = 0.046 and *p* = 0.043, respectively, for both parameters). After 24 months on suppressive cART, no differences were found in monocyte subsets or in shedding monocyte frequencies between groups (**Table 5**). However, LR-subjects showed significantly higher levels of CD11b in classical monocytes (*p* = 0.022), as well as tendencies toward higher levels of CD16, CD11b, and PDL1 in different monocyte subsets.

**Table 5:** Follow-up analysis of monocyte subsets and functional molecules (24-months of suppressive cART).

	LR-Subjects (6)			HR-Subjects (5)			
	Before cART	Post cART	*pW*	Before cART	Post cART	*pW*	*pM-W*
**% Classical monocytes**	85.3 [74.5–91.8]	81.1 [60.0–91.8]	0.3	78.8 [69.0–85.4]	80.1 [72.2–87.5]	0.5	0.9
**MFI CD11b**	2,285 [2,154–2,806]	2298 [2,028–2,785]	0.5	2,399 [1,371–2,883]	1,797 [1,142–1,977]	*0.08*	**0.022**
**% CCR2**	82.7 [66.6–92.7]	93.4 [86.3–97.5]	**0.046**	81.4 [78.9–84.9]	93.1 [90.8–95.1]	**0.043**	0.7
**% *β*7**	18.3 [4.3–32.2]	13.8 [4.2–25.4]	0.6	4.8 [2.1–52.1]	9.4 [1.8–37.1]	0.5	0.9
**% IDO**	1.0 [0.7–4.1]	0.2 [0.1–1.4]	**0.028**	1.0 [0.9–7.2]	0.4 [0.1–6.3]	*0.08*	0.8
**% PDL1**	26.5 [12.8–47.3]	16.1 [5.9–54.2]	0.5	13.7 [4.1–47.0]	5.3 [2.8–22.4]	*0.08*	0.2
**% Intermediate monocytes**	8.8 [4.1–13.3]	7.0 [1.7–19.9]	0.9	11.5 [6.0–19.9]	5.1 [3.5–13.6]	*0.08*	0.9
**MFI CD16**	1,441 [700–1,677]	1,298 [730–1,653]	0.8	536 [462–1,007]	1,043 [504–1,227]	**0.043**	0.2
**MFI CD11b**	2,792 [2,369–3,178]	3,112 [2,475–4,311]	0.3	2,836 [2,031–3,019]	2,031 [1,484–2,570]	*0.138*	*0.068*
**% CCR2**	53.9 [41.6–75.2]	63.2 [61.2–75.4]	0.2	57.9 [44.7–64.8]	60.3 [51.4–86.4]	0.3	0.4
**% *β*7**	13.2 [2.8–19.8]	7.3 [2.7–19.7]	0.5	6.7 [1.9–41.1]	3.4 [1.5–18.0]	0.3	0.6
**% IDO**	3.6 [2.0–9.7]	0.5 [0.3–3.3]	**0.028**	1.9 [1.6–11.8]	0.7 [0.0–9.1]	*0.138*	0.9
**% PDL1**	46.3 [39.0–62.9]	38.3 [24.0–68.1]	0.2	24.5 [22.3–81.5]	17.9 [9.6–37.2]	*0.138*	0.2
**% Patrolling monocytes**	2.8 [0.8–8.2]	7.0 [1.6–15.8]	0.2	2.4 [1.2–9.0]	7.7 [2.4–10.0]	0.5	0.9
**MFI CD16**	2,926 [,2037–4,878]	3,122 [,2842–3,531]	0.9	1,677 [1,160–4,545]	2,128 [1,630–3,170]	0.9	*0.068*
**MFI CD11b**	980 [708–1,256]	803 [635–1,026]	0.6	795 [520–972]	582 [484–682]	*0.08*	*0.068*
**% CCR2**	9.6 [5.3–31.5]	12.7 [6.5–20.2]	0.6	20.6 [7.5–22.4]	8.3 [4.1–20.6]	0.7	0.7
**% *β*7**	6.9 [4.8–17.0]	4.7 [4.2–8.1]	*0.075*	6.9 [6.0–38.6]	5.5 [4.2–17.2]	0.7	0.5
**% IDO**	0.5 [0.3–1.7]	0.2 [0.1–0.6]	**0.046**	0.4 [0.3–2.7]	0.4 [0.2–1.5]	0.2	0.5
**% PDL1**	74.1 [61.8–86.2]	42.6 [28.7–57.0]	**0.046**	47.4 [29.2–65.8]	18.3 [10.7–35.9]	**0.043**	*0.068*
**% Shedding monocytes**	3.5 [1.3–5.3]	5.0 [4.2–5.7]	**0.028**	4.8 [3.8–7.1]	5.1 [4.3–7.6]	0.5	0.8

Variables are expressed as median and interquartile range [IQR]. pW, Wilcoxon rank test was used to analyze longitudinal comparisons. pM-W, Mann–Whitney U test was used for transversal comparisons of post cART data. Statistically significant values are highlighted in bold, p < 0.005.

### MDSCs and Monocytes Were Directly Associated With Proinflammatory Biomarkers

**Figure 3:**
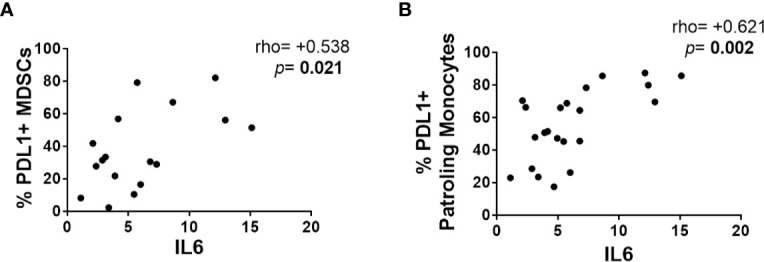
Relationships between MDSCs and monocytes with proinflammatory biomarkers. Correlations using the Spearman rank test were explored between **(A)** the frequency of PDL1^+^ MDSCs and IL-6 levels and **(B)** the frequency of PDL1^+^ patrolling monocytes and IL-6 levels.

We also explored potential correlations between MDSCs and monocyte subsets, with the levels of soluble inflammatory biomarkers, including IL-6, hsCRP, D-dimers, sCD14, LPS, IP-10, and hyaluronan. The cellular Th17/Treg ratio and the anti-CMV antibodies titers were also tested for potential associations with innate subsets. The LR-subjects included herein showed significantly higher IL-6 levels (*p* = 0.049) and trends toward higher levels of hyaluronan (*p* = 0.07) (**Supplementary Table 1**). All the associations with MDSC-related parameters are detailed in (**Supplementary Table 2**). The frequencies of CCR2^+^ MDSCs were positively associated with most of the soluble biomarkers, whereas the frequencies of PDL1^+^ MDSCs were only associated with IL-6 (**Figure 3A**). Interestingly, total-MDSCs and m-MDSCs negatively correlated with surrogate markers of microbial translocation, with D-dimers and with the Th17/Treg ratio. Regarding monocyte subsets (**Supplementary Table 3**), PDL1^+^ patrolling monocytes were strongly associated with IL-6 levels (**Figure 3B**), whereas MFI CD16 on intermediate monocytes did with Th17/Treg ratio and LPS. Globally, CCR2^+^ classical monocytes, IDO^+^ intermediate monocytes, and PDL1^+^ patrolling monocytes were the subsets more related with inflammatory markers and microbial translocation. Interestingly, while no associations were found between anti-CMV titers and monocyte markers in the total cohort, we found associations between anti-CMV titers and MFI CD11b in patrolling monocytes (rho = 0.745; *p* = 0.013), *β*7-positive patrolling monocytes (rho = 0.874; *p* = 0.001) and total frequencies of patrolling monocytes (rho = −0.773; *p* = 0.005) in LR-subjects but not in HR-subjects (**Supplementary Figure 2**).

## Discussion

We observed increased frequencies of total-MDSCs and m-MDSCs, although no differences in the distribution of monocyte subsets, preceding the poor CD4 T-cell recovery of immunodiscordant subjects. An altered expression of several functional markers, such as CCR2, CD16, CD11b, and PDL1, on MDSCs and monocyte subsets also preceded such anomalous response to cART. Remarkably, these alterations were associated with the levels of inflammatory biomarkers and the Th17/Treg ratio. Finally, longitudinal assessment showed only slight modifications on these parameters after 24 months of suppressive cART.

In recent years, MDSCs have been explored in the context of viral infections, and increased frequencies have been described in HIV-infected subjects (6). Agrati et al. have now described an expansion of MDSCs in a cohort of primary HIV infection that persisted even after one year of cART (10). Interestingly, since post-treatment MDSCs values inversely correlated with CD4 T-cell counts, authors hypothesized that MDSC could have a role in immune recovery (10). Our data support this hypothesis since we found increased frequencies of total-MDSCs and m-MDSCs in immunodiscordant subjects preceding their poor CD4 T-cell recovery in response to cART, as an early immune alteration, although these differences were not maintained after 2 years of suppressive treatment. IL-6 has been identified as one of the main drivers of MDSC expansion in HIV infection (23). In accordance, immunodiscordant subjects in our baseline cohort also showed higher levels of IL-6, which correlated with functional markers in MDSCs, mainly CCR2 and PDL1. However, our results cannot discriminate if a further reduction of CD4 cells would lead to a relative increase in MDSCs or if a further reduction of CD4 cells increases risk of bacterial infection, such as in the gut mucosa, which could result in active inflammation including IL-6 production and generation of myeloid cells including MDSCs.

MDSCs could contribute to changes in CD4 T cells and Tregs by different mechanisms. Among others, they express several enzymes and molecules with suppressive function, such as IDO, and ARG1 (both depriving key nutrition factors for T-cells from the microenvironment as L-tryptophan and L-arginine, respectively); MDSCs also produce reactive nitrogen and oxygen species and upregulate immune checkpoints, as PD-Ll and galectin-9 (23). Consequently, MDSCs are able to dampen the functions of T-cells in HIV-infected subjects (7). These cells can also act as antigen-presenting cells and favor the generation and expansion of Tregs, which requires cell-to-cell contact (24). In accordance, the frequency of total-MDSCs and m-MDSCs inversely correlated with the Th17/Treg ratio. This phenomenon could involve antigen presentation through processes of homeostatic proliferation (HP) as it has been described both in animal and human models (25, 26). Along these lines, we have recently reported increased frequencies of Tregs (19) as well as other HP surrogate markers in this cohort of immunodiscordant subjects before cART (18).

The potential alterations of monocyte subsets in immunodiscordant subjects have been scarcely investigated and never before the onset of cART (27). In the three cohorts of subjects with an incomplete immune recovery studied after cART so far, increased frequencies of intermediate monocytes have been observed (15–17). However, our cohort of immunodiscordant subjects did not show higher frequency of intermediate monocytes after the response to cART, although our limited post-treatment data could explain this contradictory result. Nevertheless, we neither observed it before the cART onset indicating that, even if this could be a post-cART characteristic of immunodiscordant subjects, this is not a baseline characteristic. There was a lack of statistical significance for the expression of activation markers in monocyte subsets, although the observed trends are consistent with a higher expression of CD11b, PDL-1 and CD16 in several monocyte subsets preceding the immunodiscordance, as well as with being a feature of immunodiscordant subjects after treatment. Curiously, we found tendencies toward reduced levels of CCR2 but increased levels of CD11b in patrolling monocytes preceding immunodiscordance. CCR2 is the major receptor for CCL2, which is one of the major inflammatory chemokines, demonstrating a potent role as a monocyte and T-cell chemoattractant (28). CD11b is a member of the *β*2-integrin family involved in monocyte activation, adhesion, and transmigration (29). Bacterial compounds such as LPS can downregulate CCR2 (30) whereas upregulate CD11b (27) in human monocytes. Consequently, our data would potentially suggest a higher microbial translocation in immunodiscordant subjects not only after cART (31), but also preceding their response to cART. However, in our baseline settings, LPS and sCD14 levels, two surrogate markers of microbial translocation, were similar between groups. Certainly, these two markers may underestimate microbial translocation, as we did not measured any marker of gram-positive bacteria translocation, such as lipoteichoic acid (LTA), which is also relevant for HIV immunopathogenesis (32). Alternatively, not only LPS but also cytomegalovirus (CMV) can be sensed by monocytes through TLR7 and TLR9, and increased anti-CMV IgG levels have been previously observed in immunodiscordant subjects (33). Nevertheless, we did not observe differences in anti-CMV IgG titers between LR- and HR-subjects at cART initiation in our cohort. However, we found that anti-CMV IgG levels were strongly associated with activation and homing markers in monocyte subsets in the LR-subjects, which could denote a higher active replication of CMV virus and its sensing by monocytes. Interestingly, in a cohort of non-HIV subjects, such titers were correlated with the expression of activation markers in monocyte subsets (34).

Monocyte and MDSC alterations contribute to HIV pathogenesis (6, 13), and thus, it is of pivotal importance to elucidate whether suppressive cART is able to impact and restore their alterations. In our cohort, we performed valuable longitudinal comparisons; however, results must be cautiously interpreted due to the limitation in post-treatment data. Although cART was able to modify the expression of several monocyte markers, such as PDL1 and CCR2, after 24 months of suppressive cART, immunodiscordant subjects still showed increased levels of the activation-related marker CD11b in all monocyte subsets and the suppression-related markers PDL1 and CD16 in patrolling monocytes. These alterations could all be related to a) the persistence of a higher Treg frequency (35), b) the irreversible damage to the intestinal mucosal integrity (36), and/or c) the exacerbated bacterial translocation reported in these subjects after cART (31). Regarding MDSCs, there is still controversy about the effect of cART in the general context of HIV infection, from a reduction (7, 37) to a full normalization (38) but even no variation after one year of cART, with persistence of the early expanded levels (10). Interestingly, we found that two years of suppressive cART was able to modify MDSC parameters in immunodiscordant subjects, making them comparable to their controls after suppressive treatment. However, we cannot conclude whether a complete normalization of values occur in both groups, since we did not test a healthy group in our setting.

Our study has limitations. First, this is an exploratory and descriptive analysis of multiple variables using small-sized groups, but our rough observations raise interesting new questions about immunodiscordance-related mechanisms that need to be addressed in higher cohorts. Indeed, the information here provided only shows associations but not a causal relationship of MDSCs with recovery. Second, although baseline CD4 T-cell counts were matched in initial groups of study, a trend toward lower CD4 T-cell counts at cART initiation was found in the restricted group of immunodiscordant subjects herein included, as this is a potent risk factor for immunodiscordance (2). However, adjusted analyses for CD4 T-cell counts proved that our results were not biased by this parameter. Nevertheless, it cannot be excluded that the poor CD4 recovery in the LR group could be the result of further too low baseline CD4 counts and further advance of immunodeficiency. Finally, we were not able to determine the frequencies of g-MDSCs due to their extremely low number of events which prevented us from reaching any conclusion about this MDSC population. The extremely low frequencies of g-MDCS in non-cancer could probably explain the difficulty of determining them in this study. Globally, our data contribute to a better understanding of the mechanisms preceding immunodiscordance to cART, which seem to involve an early immunosuppressive environment.

## Conclusion

Importantly, before the onset of cART, subjects with subsequent poor CD4-recovery showed expanded MDSCs and both innate subsets, MDSCs and monocytes showed altered expression of functional markers as CCR2, CD16, CD11b, and PDL1. Moreover, two years of suppressive cART scarcely modified these parameters in the groups of study. Innate subsets correlated with soluble biomarkers of inflammation and bacterial translocation with the Th17/Treg ratio and anti-CMV titers. Our study contributes to the knowledge of the main immune alterations preceding poor CD4-recovery. Such knowledge can be useful in the search for new therapeutic targets for this clinical situation.

## Data Availability Statement

The raw data supporting the conclusions of this article will be made available by the authors, without undue reservation.

## Ethics Statement

The studies involving human participants were reviewed and approved by Comité de Etica de la Investigación de los Hospitales Universitarios Virgen Macarena-Virgen del Rocío. The patients/participants provided their written informed consent to participate in this study.

## Author Contributions

IR-S designed the study, performed experiments, data analysis and interpretation, and wrote the draft. RP-B contributed to the design of the study and revised the draft for important intellectual content. JG, SM, DV, and VE provided samples and critically revised the draft. MM-F, AR, FV, and ML contributed to the study design and critically revised the draft. YP conceived and designed the study, contributed to data analysis and interpretation and to the writing. All authors contributed to the article and approved the submitted version.

## Funding

This study was funded by grants from the Instituto de Salud Carlos III, Fondo de Investigación Sanitaria [FIS; PI16/00503, PI18/01216, PI19/01337; PI20/00326] and contratos para la intensificación de la actividad investigadora en el SNS [INT20/00031], co-funded by European Regional Development Fund/European Social Fund; “A way to make Europe”/”Investing in your future”, by the Programa de Suport als Grups de Recerca AGAUR (2017SGR948). The Spanish AIDS Research Network of Excellence also supported this study (RD16/0025/0019; RD16/0025/0006). YP was supported by the Consejería de Salud y Bienestar Social of Junta de Andalucía through the ‘‘Nicolás Monardes’’ programme [C-0013-2017]. AR is supported by IISPV through the project “2019/IISPV/05” (Boosting Young Talent), by GeSIDA through the “III Premio a Jóvenes Investigadores” and by ISCIII through the Miguel Servet Program “CP19/00146”. The funders had no role in study design, data collection and interpretation, or the decision to submit the work for publication.

## Conflict of Interest

The authors declare that the research was conducted in the absence of any commercial or financial relationships that could be construed as a potential conflict of interest.

## Supplementary Material

Supplementary Figure 1 Representative gating strategy of MDSCs and monocytes subsets.

Supplementary Figure 2 Associations between CMV titers and patrolling monocytes. CMV titters were strongly associated with homing and activation markers of patrolling monocytes, but only in LR-subjects. Correlations were performed in 11 LR-subjects and 14 HR-subjects.

## Abbreviations

cART, combined antiretroviral therapy; CMV, Cytomegalovirus; HIV, Human Immunodeficiency Virus; HP, homeostatic proliferation; hsCRP, high-sensitivity C-reactive protein; MDSC, myeloid-derived suppressor cells; PBMC, peripheral blood mononuclear cells; Treg, regulatory T-cells.
