# Supplementary material for: Increased Frequencies of Myeloid-Derived Suppressor Cells Precede Immunodiscordance in HIV-Infected Subjects
Source: Front Immunol. 2020 Nov 6;11:581307. doi: 10.3389/fimmu.2020.581307 (PMC7677300; doi:10.3389/fimmu.2020.581307)
Supplement: Supplementary Figure 1 — Representative gating strategy of MDSCs and monocytes subsets. [file DataSheet_1.docx]

**Supplementary Material**

**Supplementary Methods**

Antibodies used were: anti-CD3 APC-H7, anti-CD4 BV687, anti-CD25 BV605, anti-FoxP3 PE-CF594, anti-CD3 FITC, anti-CD19 FITC, anti-CD20 FITC, anti-PDL1 PE-CF594, anti-CD14 BV650, anti-HLA-DR BV711, anti-CD15 V450, anti-CD16 BV786, anti-β7 integrin APC, anti-CD33 AF700, anti-CD11b APCH7 (BD Bioscience, USA), anti-CCR2 BV605 (Biolegend, USA), anti-IDO PE, anti-IL17A APC (eBioscience, USA). Isotype controls were used for determine the expression of PDL1, CD16, β7, CCR2, IDO, CD25 and Foxp3.

**Supplementary Table 1**: Levels of soluble biomarkers of inflammation and Th17/Treg ratio before cART initiation.

|  | LR-subjects  (12) | HR-subjects  (14) | p |
| --- | --- | --- | --- |
| IL6 (pg/mL) # | 8.7 [3.1-13.0] | 4.8 [3.1-6.2] | **0.049** |
| hsCRP (mg/L) # | 6.4 [0.7-9.7] | 2.4 [0.8-3.9] | 0.188 |
| D-dimers (ug/L) | 419 [305-1095] | 478 [273-828] | 0.9 |
| sCD14 (ng/mL) | 3.6 [3.4-3.8] | 3.5 [3.4-3.7] | 0.5 |
| LPS (EU/mL) | 0.2 [0.13-0.22] | 0.2 [0.13-.27] | 0.8 |
| CMV (AU/mL) | 1004 [419-1511] | 1090 [466-1515] | 0.7 |
| IP-10 (pg/mL) | 1278 [420-1834] | 710 [445-1025] | 0.2 |
| Hyaluronan (ng/mL) | 33.1 [20.4-90.0] | 19.0 [12.1-25.1] | *0.072* |
| Th17/Treg ratio #* | 0.7 [0.1-8.8] | 0.8 [0.1-3.1] | 0.8 |

Variables are expressed as median and interquartile range [IQR]. Mann–Whitney *U* test was used for comparisons. #One outlier value was excluded from these variables. *n=8 (LR) and n=9 (HR).

**Supplementary Table 2**: Correlations between MDSCs and cellular markers with proinflammatory biomarkers and Th17/Treg ratio before cART initiation.

|  | **IL6 #** | **hsCRP #** | **D-dimers** | **sCD14** | **LPS** | **CMV** | **IP-10** | **Hyaluronan** | **Th17/Treg#*** |
| --- | --- | --- | --- | --- | --- | --- | --- | --- | --- |
| **% total-MDSCs** | NS | NS | NS | **rho= -0.396**  ***p*= 0.045** | NS | NS | rho= 0.286  *p*= 0.166 | NS | **rho= -0.483**  ***p*= 0.050** |
| **% m-MDSCs** | NS | NS | NS | rho= -0.386  *p*= 0.051 | NS | NS | NS | NS | **rho= -0.596**  ***p*= 0.012** |
| **% CCR2^+^ MDSCs*** | **rho= 0.591**  ***p*= 0.010** | **rho=0.521**  ***p*= 0.027** | rho= **0.517**  *p*= **0.028** | NS | rho= 0.434  *p=* 0.072 | NS | rho= 0.368  *p*= 0.121 | **rho= 0.599**  ***p*= 0.020** | NS |
| **% β7^+^ MDSCs*** | NS | NS | NS | NS | NS | NS | NS | NS | NS |
| **% IDO^+^ MDSCs*** | NS | NS | NS | NS | NS | NS | NS | rho= -0.345  *p*= 0.175 | NS |
| **% PDL1^+^ MDSCs*** | **rho= 0.538**  ***p*= 0.021** | NS | NS | NS | NS | NS | NS | NS | NS |

Correlations were assessed using Spearman rank test (n=25). NS, non-significative (data is only given when p≤0.2). Correlations between functional markers in MDSCs and proinflammatory biomarkers were performed in 19 subjects. #One outlier value was excluded from these variables. *n=17

S**upplementary Table 3**: Correlations between monocytes subsets and proinflammatory biomarkers.

|  | **IL6 #** | **hsCRP #** | **D-dimers** | **sCD14** | **LPS** | **CMV** | **IP-10** | **Hyaluronan** | **Th17/Treg**# |
| --- | --- | --- | --- | --- | --- | --- | --- | --- | --- |
| **% Classical monocytes** | NS | NS | NS | rho= 0.264 *p*= 0.192 | NS | NS | NS | NS | NS |
| **MFI CD11b** | NS | NS | NS | NS | NS | NS | NS | NS | rho= -0.402  *p*= 0.110 |
| **% CCR2** | rho= 0.321  *p*= 0.117 | rho= 0.345  *p*= 0.091 | rho= 0.376  *p*= 0.070 | rho= 0.292 *p*= 0.148 | NS | NS | NS | **rho= 0.413 *p*= 0.045** | rho= 0.363  *p*= 0.152 |
| **% β7** | NS | NS | NS | NS | NS | NS | NS | NS | NS |
| **% IDO** | rho= 0.286  *p*= 0.166 | NS | NS | NS | NS | NS | rho= 0.335 *p*= 0.101 | rho= 0.290  *p*= 0.169 | NS |
| **% PDL1** | rho= 0.352  *p*= 0.085 | NS | NS | NS | NS | NS | NS | NS | NS |
| **% Intermediate monocytes** | NS | NS | NS | NS | NS | NS | NS | NS | NS |
| **MFI CD16** | NS | NS | NS | NS | **rho=-0.421**  ***p*= 0.041** | NS | NS | NS | **rho= -0.588**  ***p*= 0.013** |
| **MFI CD11b** | rho= 0.257  *p*= 0.2 | NS | NS | NS | NS | NS | NS | NS | rho= -0.358  *p*= 0.158 |
| **% CCR2** | NS | NS | NS | NS | NS | NS | rho= -0.265 *p*= 0.2 | NS | NS |
| **% β7** | NS | NS | NS | NS | NS | NS |  | NS | NS |
| **% IDO** | rho= 0.307  *p*= 0.136 | NS | NS | **rho=0.398**  ***p*= 0.044** | NS | NS | rho= 0.364 *p*= 0.073 | rho= 0.361 *p*= 0.083 | NS |
| **% PDL1** | rho= 0.279  *p*= 0.176 | NS | NS | NS | NS | rho= -0.295 *p*= 0.152 | NS | NS | NS |
| **% Patrolling monocytes** | rho= -0.382  *p*= 0.059 | rho= -0.318  *p*= 0.121 | **rho= -0.441 *p*= 0.031** | NS | NS | rho= -0.338 *p*= 0.099 | NS | rho= -0.294 *p*= 0.163 | NS |
| **MFI CD16*** | NS | NS | NS | NS | rho= -0.338  *p*= 0.124 | rho= -0.304 *p*= 0.158 | NS | NS | rho= -0.446  *p*= 0.073 |
| **MFI CD11b*** | rho= 0.275  *p*= 0.2 | NS | NS | NS | NS | NS | NS | NS | **rho= -0.674**  ***p*= 0.003** |
| **% CCR2*** | NS | NS | NS | NS | NS | NS | NS | NS | NS |
| **% β7*** | NS | NS | NS | NS | NS | rho= 0.382 *p*= 0.072 | NS | NS | **rho= -0.512**  ***p*= 0.036** |
| **% IDO*** | rho= 0.323  *p*= 0.133 | NS | NS | NS | rho= 0.290 *p*= 0.190 | rho= 0.3838 *p*= 0.115 | rho= 0.277 *p*= 0.2 | NS | NS |
| **% PDL1*** | **rho= 0.621**  ***p*= 0.002** | NS | NS | NS | NS | NS | NS | **rho= 0.523 *p*= 0.012** | rho= -0.362  *p*= 0.169 |
| **% Shedding monocytes*** | NS | NS | NS | NS | rho= 0.295 *p*= 0.182 | NS | NS | NS | NS |

Correlations were assessed using Spearman rank test (n=25). NS, non-significative (data is only given when p≤0.2). *Correlations in patrolling monocytes and shedding monocytes were performed in 23 subjects. #One outlier value was excluded from these variables. Correlations with Th17/Treg ratio were performed in 17 subjects.

**Supplementary Figures**

Supplementary Figure 1.

**
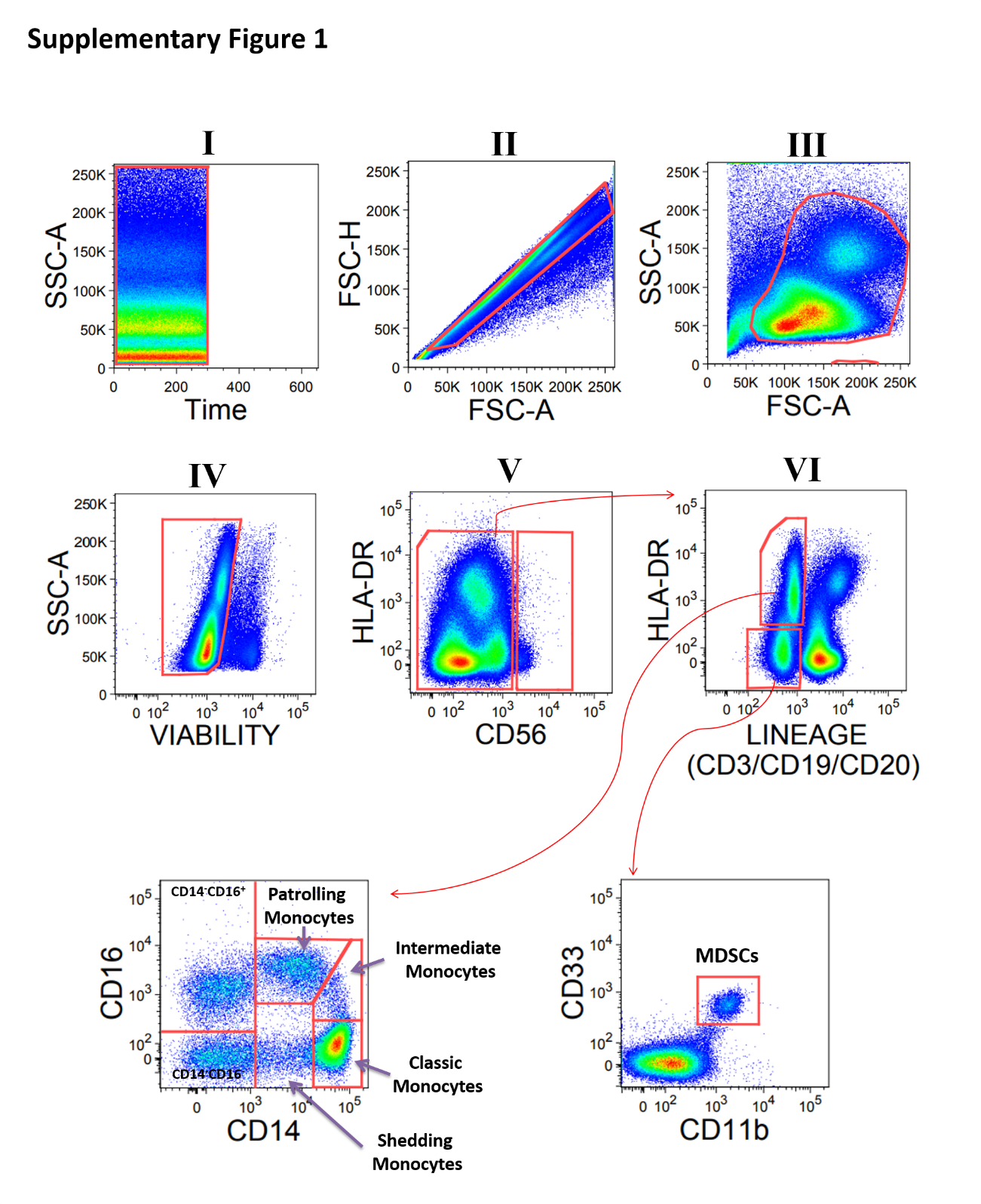
**

Supplementary Figure 2.
